# Disrupting VEGF-A paracrine and autocrine loops by targeting SHP-1 suppresses triple negative breast cancer metastasis

**DOI:** 10.1038/srep28888

**Published:** 2016-07-01

**Authors:** Jung-Chen Su, Ai-Chung Mar, Szu-Hsien Wu, Wei-Tien Tai, Pei-Yi Chu, Chia-Yun Wu, Ling-Ming Tseng, Te-Chang Lee, Kuen-Feng Chen, Chun-Yu Liu, Hao-Chieh Chiu, Chung-Wai Shiau

**Affiliations:** 1 Institute of Biopharmaceutical Sciences, National Yang-Ming University, Taipei, Taiwan; 2 Department of Clinical Laboratory Sciences and Medical Biotechnology, National Taiwan University, Taipei, Taiwan; 3 Taiwan International Graduate Program in Molecular Medicine, National Yang-Ming University and Academia Sinica, Taipei 11529, Taiwan; 4 Department of Medical Research, National Taiwan University Hospital, Taipei, Taiwan; 5 National Center of Excellence for Clinical Trial and Research, National Taiwan University Hospital, Taipei, Taiwan; 6 School of Medicine, College of Medicine, Fu-Jen Catholic University, New Taipei City, Taiwan; 7 Department of Pathology, Show Chwan Memorial Hospital, Changhua City, Taiwan; 8 Division of Medical Oncology, Department of Oncology, Taipei Veterans General Hospital, Taipei, Taiwan; 9 School of Medicine, National Yang-Ming University, Taipei, Taiwan; 10 Department of Surgery, Taipei Veterans General Hospital, Taipei, Taiwan; 11 Institute of Biomedical Sciences, Academia Sinica, Taipei 11529, Taiwan

## Abstract

Patients with triple-negative breast cancer (TNBC) had an increased likelihood of distant recurrence and death, as compared with those with non-TNBC subtype. Regorafenib is a multi-receptor tyrosine kinase (RTK) inhibitor targeting oncogenesis and has been approved for metastatic colorectal cancer and advanced gastrointestinal stromal tumor. Recent studies suggest regorafenib acts as a SHP-1 phosphatase agonist. Here, we investigated the potential of regorafenib to suppress metastasis of TNBC cells through targeting SHP-1/p-STAT3/VEGF-A axis. We found a significant correlation between cancer cell migration and SHP-1/p-STAT3/VEGF-A expression in human TNBC cells. Clinically, high VEGF-A expression is associated with worse disease-free and distant metastasis-free survival. Regorafenib induced significant anti-migratory effects, in association with downregulation of p-STAT3 and VEGF-A. To exclude the role of RTK inhibition in regorafenib-induced anti-metastasis, we synthesized a regorafenib derivative, SC-78, that had minimal effect on VEGFR2 and PDGFR kinase inhibition, while having more potent effects on SHP-1 activation. SC-78 demonstrated superior *in vitro* and *in vivo* anti-migration to regorafenib. Furthermore, VEGF-A dependent autocrine/paracrine loops were disrupted by regorafenib and SC-78. This study implies that SHP-1/p-STAT3/VEGF-A axis is a potential therapeutic target for metastatic TNBC, and the more potent SC-78 may be a promising lead for suppressing metastasis of TNBC.

Triple negative breast cancer (TNBC) is characterized by more aggressive disease behavior and higher probability of distant metastases, preferentially to the lung, bones and brain^1^,^2^,^3^,^4^. In addition, TNBC per se has also been shown as a factor indicative of aggressive metastatic breast cancer^3^,^5^. Patients with metastatic TNBC have also been reported to have a poorer outcome; Kobayashi *et al.*^6^ reported a median survival time of 18.6 months, and similarly Zhang *et al.*^7^ reported a median survival time of 19.7 months in patients with metastatic TNBC. Therefore, there is an urgent and unmet need to further understand the molecular mechanisms and tumor biology of TNBC and develop more effective targeted therapy to improve outcomes in TNBC patients.

Epithelial dysregulation and microenvironment accommodation has been shown to play a vital role in tumor metastasis. The factors secreted by tumor cells and tumor-associated stromal cells enhance metastatic seeding of circulating tumor cells. Among tumor secretory factors, vascular endothelial growth factor A (VEGF-A) is highly correlated with tumor progression, invasion, and angiogenesis in TNBCs and other malignancies^8^,^9^,^10^,^11^. Disruption of VEGF-induced tumor metastasis and angiogenesis may provide an effective therapeutic option for TNBC patients. Indeed, the addition of bevacizumab, an anti-VEGF monoclonal antibody, has been consistently shown to increase overall response rate and progression-free survival (PFS) in several large phase III randomized trials in patients with metastatic TNBC, despite there is no significant survival benefit on overall survival (OS)^12^,^13^,^14^. Nevertheless, guidelines from the U.S. National Comprehensive Cancer Network have included the combination of bevacizumab with paclitaxel as an option for patients with metastatic breast cancer^15^. Recent guidelines from the American Society for Clinical Oncology has defined a selected population of metastatic breast cancer patients as those involving life-threating disease or severe symptoms, for which they recommend adding bevacizumab to single-agent chemotherapy^16^.

In addition to the most studied bevacizumab, several types of antiangiogenic agents have been studied in patients with breast cancer, and many of these agents are approved for the treatment of other malignancies such as renal cell cancer, colorectal cancer (CRC), ovarian cancer and others. These include antibodies such as ramucirumab (that binds to the VEGF receptor [VEGFR]), VEGFR-mimetics such as aflibercept, and small molecule multi-targeted oral tyrosine kinase inhibitors (TKIs) that inhibit a wide variety of targets, including VEGFR, platelet-derived growth factor receptor (PDGFR), and many others^17^. Regorafenib, a multi-kinase inhibitor against Raf, RET, PDGFR, VEGFR and so on^18^, is a diphenyl urea compound and has shown anti-tumor effects in preclinical studies against various cancers^18^. Phase III studies of regorafenib showed moderate improvement on OS in metastatic CRC patients^15^,^17^.

The regulation of VEGF expression occurs at multiple levels^19^, and signal transducer and activator of transcription 3 (STAT3) directly regulates VEGF through transcription^20^,^21^. Moreover, a protein tyrosine phosphatase, SHP-1, has been reported to negatively regulate STAT3 in several types of cancers^22^,^23^. SHP-1 (SH2 domain-containing phosphatase 1) is a nonreceptor protein tyrosine phosphatase (PTP) that has been reported to negatively regulate hematopoietic cancer cell proliferation, and function as a tumor suppressor. The crystal structure study indicated that SHP-1 contains two SH-2 domain (N-SH2 and C-SH2) and one catalytic PTP domain^24^. The activity of SHP-1 strongly depends on the conformational rearrangement through ligand or chemical binding. The interaction between ligands/chemicals and SH2 domains will induce conformational changes of SHP-1, resulting in the releasing of the N-SH2 domain from the PTP domain and initiating the whirling of the C-SH2 domain and further exposing the active site of PTP domain^25^,^26^,^27^. Interestingly, we previously reported that regorafenib could directly activate SHP-1 in HCC and CRC, which was independent of its angio-kinase inhibition^28^,^29^.

In this study, we dissected the relationship between regorafenib, tumor microenvironment, and tumor metastasis. We found that regorafenib suppressed metastatic potential of TNBC cells though SHP-1/p-STAT3/VEGF-A dependent mechanism. Furthermore, a regorafenib derivative, SC-78, with minimal effect on VEGFR2 and PDGFR kinase inhibition, showed more potent effects than regorafenib on suppressing TNBC cells metastasis *in vivo*.

## Results

### Correlation between the SHP-1/p-STAT3/VEGF-A axis and tumor metastasis

**Figure 1:**
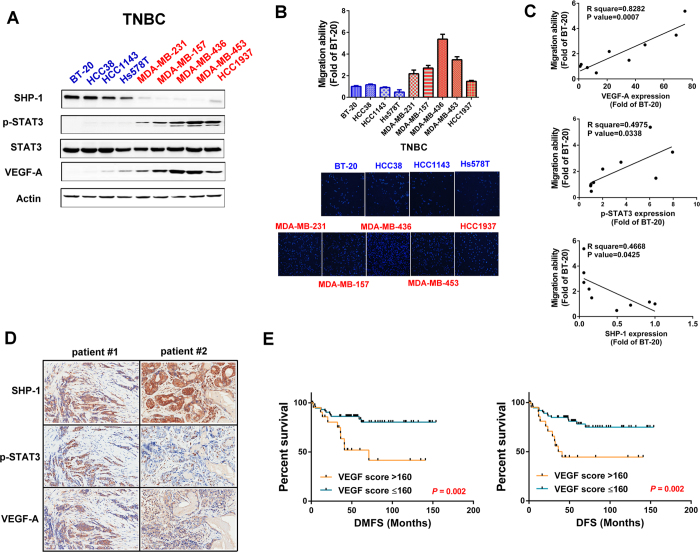
SHP-1 is negatively associated with p-STAT3/VEGF-A signaling and metastasis in TNBC cells and clinical samples. (**A**) Protein expression pattern of SHP-1, p-STAT3, and VEGF-A in nine TNBC cell lines were analyzed by western blot. (**B**) The migration abilities of nine TNBC cell lines were analyzed by Transwell assay. DAPI stains the nuclei. BT-20 cells were used as a normalization control. (**C**) The correlation (linear regression model) of VEGF-A (*top*), p-STAT3 (*middle*), and SHP-1 (*bottom*) and migration ability in nine TNBC cell lines. (**D**) TNBC cells from representative two patients with SHP-1, p-STAT3, and VEGF-A staining. (200×) (**E**) Kaplan-Meier graph was prepared to compare DMFS (*left*) and DFS (*right*) in patients with high VEGF-A (H score > 160) or low VEGF-A (H score < = 160) levels for the indicated time of follow up. Chi-square test indicated a significant difference between VEGF-A high (N = 21) and low (N = 76) patients.

In patients acquiring breast tumors, higher VEGF expression is correlated with poor survival outcome^30^,^31^. However, the correlation between SHP-1, p-STAT3 and VEGF-A in TNBC cells remains unknown. To determine the association of the SHP-1/p-STAT3/VEGF-A cascade and tumor metastatic ability, we analyzed the protein expression of SHP-1, p-STAT3, and VEGF-A in several TNBC cell lines. Cells exhibiting higher SHP-1 protein expressions have lower p-STAT3 and VEGF-A levels (Fig. 1A). In addition, the migration ability of TNBC cells is positively correlated with p-STAT3 and VEGF-A, but inversely correlated with SHP-1 expressions (Fig. 1B,C). These results suggest that the metastatic ability of TNBC cells might be related to the SHP-1/p-STAT3/VEGF-A cascade. We also performed immunohistochemistry (IHC) assay to examine the levels of these three proteins in primary breast tumor samples from a cohort of 97 patients with TNBC. The American Joint Committee on Cancer (AJCC) tumor stage, and clinical outcome of patients according to higher or lower VEGF-A expression based on a histology score (H-score) cut-off at 160. Cox regression analysis revealed that higher VEGF-A expression correlated with worse outcome (in particular distant metastasis), independent of tumor stage (Supplementary Table S1). IHC staining found some representative TNBC patient samples showing strong SHP-1 staining correlated with low levels of p-STAT3 and VEGF-A, and vice versa (Fig. 1D). To further investigate VEGF-A levels and patient outcomes, Kaplan-Meier curves were analyzed for the risk of developing metastasis in TNBC patients according to those with high or low VEGF-A levels. This analysis revealed that the distant metastasis-free survival (DMFS, Fig. 1E, left), and disease-free survival (DFS, Fig. 1E, right) and specific response to the expression of VEGF-A in the patients with TNBC tumors. In addition, we also performed in silico analysis using a publically available database Kaplan-Meier Plotter. Kaplan-Meir survival analysis of VEGF-A mRNA in 249 TNBC patients revealed that DFS was shorter in the VEGF-A mRNA high expression group than in the VEGF-A mRNA low expression group (Supplement Figure S1). Taken together, these findings indicated that SHP-1 negatively regulates p-STAT3/VEGF-A and inhibits metastasis in TNBC cells.

### Regorafenib abolishes TNBC cell migratory effect and blocks p-STAT3/VEGF-A signaling

**Figure 2:**
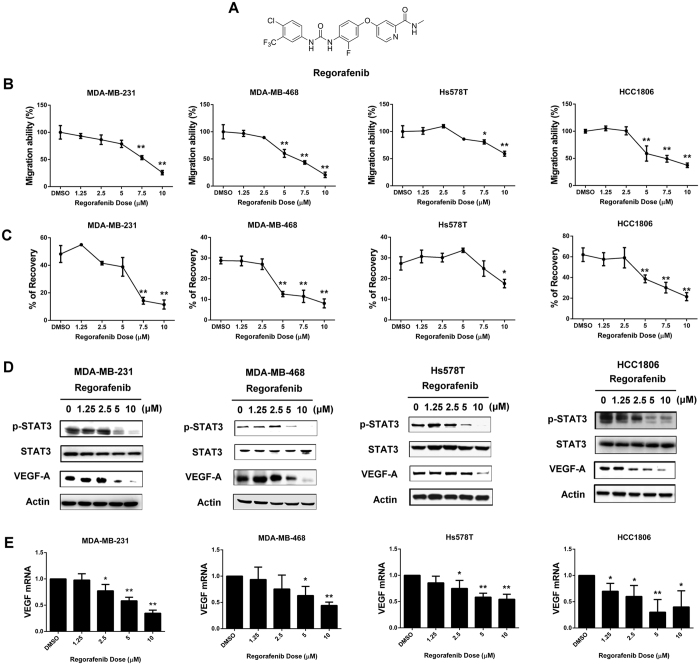
Regorafenib reduced TNBC cell migration and targeted p-STAT3/VEGF-A signaling. (**A**) Chemical structure of regorafenib. (**B,C**), Transwell assay (**B**) and wound-healing assay (**C**) were performed in TNBC cell lines after regorafenib treatment for 24 h. *p < 0.05, **p < 0.01. (**D**) dose-dependent effects of regorafenib on p-STAT3 and VEGF-A proteins were analyzed by western blot. (**E**) Dose-dependent effects of regorafenib on VEGF-A mRNA were analyzed by qPCR. TNBC cells were exposed to the indicated doses for 24 hours. *p < 0.05, **p < 0.01.

To examine the anti-cancer migratory effects of regorafenib against TNBC cells, we treated four TNBC cell lines with regorafenib, structure shown as Fig. 2A. As shown in Fig. 2B,C, regorafenib suppressed the migration of 4 TNBC cell lines. To investigate the involvement of the p-STAT3/VEGF-A pathway in regorafenib-mediated migration inhibition, we analyzed the protein expression of p-STAT3 in TNBC cells exposed to regorafenib. The protein levels of p-STAT3 were decreased in a dose dependent manner in TNBC cells after regorafenib treatment (Fig. 2D). Furthermore, we also demonstrated that the protein and mRNA levels of VEGF-A expression were reduced in TNBC cells exposed to regorafenib (Fig. 2E), implying that regorafenib might inhibit the migration of TNBC cells through decreasing p-STAT3/VEGF-A signaling.

### Regorafenib participated in SHP-1/p-STAT3/VEGF-A signaling and subsequently inhibited TNBC cell migration

**Figure 3:**
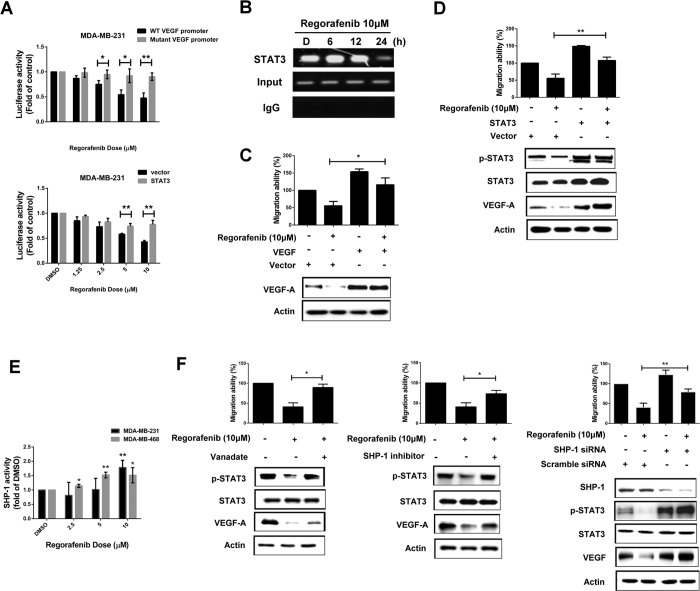
Regorafenib transcriptionally inhibited VEGF-A expression through decreasing the binding of STAT3 on the promoter of VEGF-A. (**A**) *Top*, MDA-MB-231 cells were co-transfected with a *Renilla* control vector and plasmids containing the firefly luciferase gene driven by wild-type or STAT3 binding site-mutated VEGF-A promoter. After transfection for 48 h, cells were treated with regorafenib for 24 h. Promoter activity was analyzed by luciferase assay after regorafenib treatment. *Bottom*, MDA-MB-231 cells were transfected with vector-control or STAT3-overexpression plasmid for 48 h. After that, cells were further co-transfected with *Renilla* and wild-type VEGF-A promoter and detect promoter activity as mentioned above. *p < 0.05, **p < 0.01. (**B**) After regorafenib treatment for 24 h, STAT3 binding site fragment was detected by PCR in ChIP samples precipitated with STAT3 and rabbit IgG control antibodies in MDA-MB-231 cells. (**C,D**), MDA-MB-231 cells were transfected, respectively, with control vector or VEGF-A overexpression plasmid (**C**) or STAT3 overexpression plasmid (**D**) for 48 h. After transfection, the cells were treated w/wo regorafenib for 24 h and were subjected to western blot assay or seeded to Transwell to analyze the migration ability. *p < 0.05, **p < 0.01. (**E**) Cells were treated with regorafenib at the indicated dosages for 24 h and then cell lysates were analyzed by SHP-1 phosphatase activity assay. *p < 0.05, **p < 0.01. (**F**) MDA-MB-231 cells were pretreated with pan-phosphatase inhibitor (*left*), or specific SHP-1 inhibitor (*Middle*) for 1 h before regorafenib treatment. *Right,* MDA-MB-231 cells were transfected, respectively, with control siRNA or SHP-1 siRNA for 48 h. After transfection, the cells were treated w/wo regorafenib (10 μM) for 24 h. The protein levels were analyzed by western blot assay or cells seeded to Transwell to analyze the migration ability. *p < 0.05, **p < 0.01.

To confirm that regorafenib-reduced VEGF-A is transcriptionally activated by STAT3, we analyzed the promoter activity of VEGF-A in MDA-MB-231 cells after regorafenib treatment. As shown in Fig. 3A, regorafenib dose-dependently decreased the promoter activity of VEGF. In addition, the inhibition of VEGF promoter activity by regorafenib was abolished when the binding site of STAT3 in VEGF promoter was mutated (Fig. 3A, top) or when STAT3 was overexpressed (Fig. 3A, bottom). Moreover, we further demonstrated that binding of STAT3 to VEGF promoter was decreased in MDA-MB-231 cells after regorafenib treatment (Fig. 3B). These results indicated that regorafenib transcriptionally attenuated VEGF-A expression through STAT3 signaling in TNBC cells. To further demonstrate that VEGF-A/STAT3 is involved in inhibition of regorafenib-dependent tumor migration in TNBC cells, we transfected VEGF-A or STAT3 overexpression plasmid into MDA-MB-231 cells and analyzed the migration ability of MDA-MB-231 cells after regorafenib treatment. As shown in Fig. 3C,D, the reduction in migration caused by regorafinib in MDA-MB-231 cells could be rescued after VEGF-A or STAT3 were overexpressed. In our previous studies, regorafenib inhibited the phosphorylation of STAT3 through activating SHP-1 activity; therefore, we hypothesized that regorafenib may inhibit the metastasis of TNBC cells through targeting the SHP-1/p-STAT3/VEGF-A pathway. As shown in Fig. 3E, regorafenib activated SHP-1 activity in both MDA-MB-231 and MDA-MB-468 cells. We also treated the cells with pan-phosphatase inhibitor, (Fig. 3F, *left*), specific SHP-1 inhibitor (Fig. 3F, *middle*), or si-RNA targeting SHP-1 (Fig. 3F, *right*) and determined the migration ability after regorafenib treatment in MDA-MB-231 cells. As expected, regorafenib-induced VEGF-A protein expression and migration ability inhibition were rescued by the aforementioned pre-treatment. The above results indicated that regorafenib inhibited the migration of TNBC cells through targeting SHP-1/p-STAT3/VEGF-A signaling.

### SC-78 blocking of TNBC cell migration is independent of kinase inhibition activity

**Figure 4:**
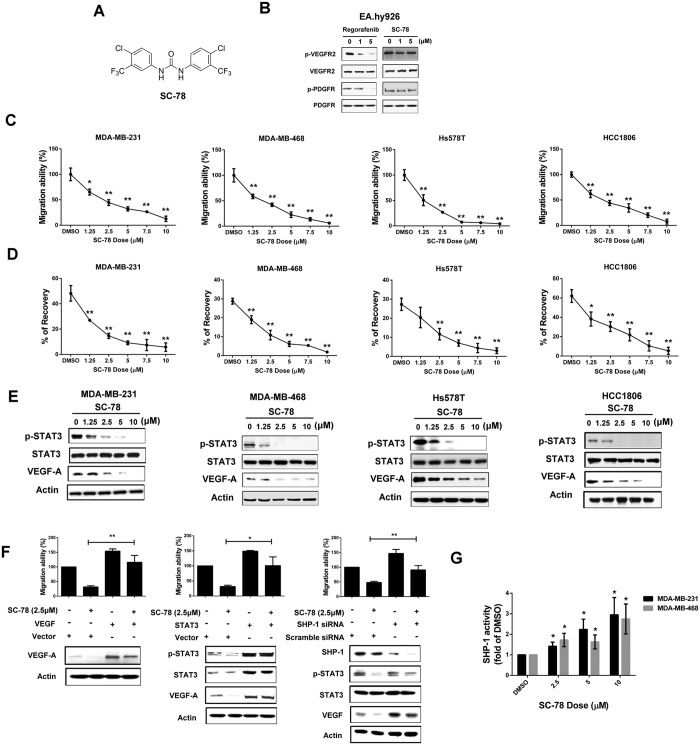
SC-78, a derivative of regorafenib, exhibited more potent anti-migratory effects than regorafenib. (**A**) Chemical structure of SC-78. (**B**) The protein levels of p-VEGFR2, VEGFR2, p-PDGFR, and PDGFR in EAhy926 treated with various doses of regorafenib or SC-78 were determined by western blot. (**C,D**), The effect of SC-78 on migration of human TNBC cells was analyzed by Transwell migration assay (**C**) and wound-healing assay (**D**). *p < 0.05, **p < 0.01. (**E**) Human TNBC cells were treated with SC-78 dose dependently for 24 h, and the cell lysates were subjected to western blot assay. (**F**) MDA-MB-231 cells were transfected with vector control or VEGF-A overexpression plasmid (*left*), STAT3 overexpression plasmid (*middle*), scramble or SHP-1 siRNA (*right*), respectively. After 48 h transfection, cells were treated with SC-78 (2.5 μM) for 24 h and subjected to western blot assay or cells analyzed by Transwell migration assay. *p < 0.05, **p < 0.01. (**G**) SHP-1 activity assay was performed in MDA-MB-231 and MDA-MB-468 cells treated with SC-78 dose dependently. *p < 0.05, **p < 0.01.

To dissect the involvement of other kinases in metastasis of TNBCs, we designed a new compound, SC-78, with a similar structure to regorafenib but without hinge binding activity in the ATP binding pocket of kinases (Fig. 4A). SC-78 displayed minimal inhibition of VEGFR-2 and PDGFR-β kinases comparing to regorafenib (Fig. 4B). However, SC-78 significantly inhibited the migration ability (Fig. 4C,D) and decreased the protein expression of p-STAT3 and VEGF-A more potently than regorafenib in human TNBC cells (Fig. 4E). To validate the anti-migration in TNBC cells by SC-78 is dependent on SHP-1/p-STAT3/VEGF-A signaling, we overexpressed VEGF-A and STAT3 in MDA-MB-231 cells and found the anti-migration by SC-78 could be rescued after ectopic expression of VEGF-A or STAT3, respectively (Fig. 4F, *left and middle*). In addition, silencing of SHP-1 abolished the inhibition of migration of MDA-MB-231 cells by SC-78 (Fig. 4F, *right*). Furthermore, SC-78 treatment increased SHP-1 activity in a dose-dependent manner in MDA-MB-231 and MDA-MB-468 cells (Fig. 4G). These results suggested that SC-78 is a more potent inhibitor of the migration of human TNBC cells than regorafenib, through specifically targeting SHP-1/p-STAT3/VEGF-A pathway.

### Regorafenib and SC-78 abolish VEGF-A secretion thus modulating both the VEGF-A-autocrine and paracrine loop

**Figure 5:**
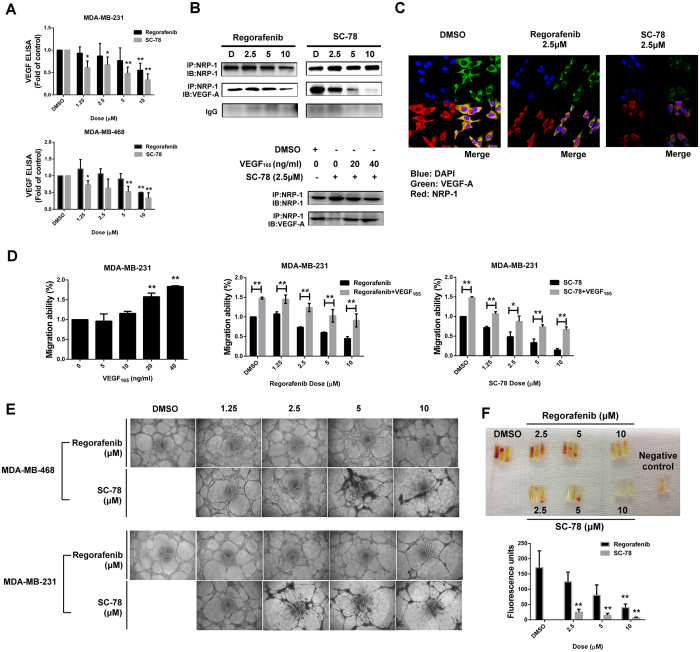
Both regorafenib and SC-78 targeted autocrine and paracrine regulation of VEGF-A in TNBCs. (**A**) Cells were treated with SC-78 or regorafenib for 24 h. After that, the medium was replaced with fresh medium and incubated for another 24 h. Then, the medium was collected and ELISA was used to measure the VEGF-A level in the CM harvested from TNBC cells w/wo treatment of SC-78 or regorafenib. *p < 0.05, **p < 0.01. (**B**) *Top*, For IP-western assay, MDA-MB-231 cells were incubated with CM for 24 h and total lysate was immunoprecipitated using anti-NRP-1 antibody, and western blotting was performed using the indicated antibodies. *Bottom*, the methods to collect CM described above were followed, and the VEGF_165_ recombinant protein was re-added into CM harvested from MDA-MB-231 cells w/wo treatment of SC-78 or regorafenib for 24 h, then cells were further analyzed by IP-western assay. (**C**) The co-localization of VEGF-A (green) and NRP-1 (red) in MDA-MB-231 cells treated with CM harvested from MDA-MB-231cells w/wo treatment of SC-78 or regorafenib was examined under a confocal microscope after immunofluorescent staining. DAPI stains the nuclei. (**D**) *Left,* MDA-MB-231 cells were seeded and treated with VEGF_165_ at the indicated concentration (upper chamber of the Transwell plate). The migrated cells were stained with DAPI and counted after 18 h by immunofluorescence microscope. MDA-MB-231 cells were exposed to regorafenib (*middle*) or SC-78 (*right*) w/wo the addition of VEGF_165_. Cells were subjected to Transwell migration assay as mentioned above. *p < 0.05, **p < 0.01. (**E**) CM was harvested from TNBC cells w/wo treatment of regorafenib or SC-78. EA.hy926 cells (5 × 10^4^) in CM were seeded and tube-formation ability was examined after incubation for 24 h. (F) For DIVAA, mice were implanted with silicone tubes containing Matrigel and supplemented with angiogenic factors and CM harvested from cells treated with various doses of regorafenib or SC-78 (n = 3). Neovascularization in the angioreactors was quantified using a spectrofluorometer 14 days after implantation. *p < 0.05, **p < 0.01.

VEGF secretion by tumor cells affects both cancer cells and peripheral endothelial cell migration, thus impairing tumor migration and angiogenesis. To further confirm that the secretion of VEGF-A was reduced by regorafenib and SC-78, we analyzed the VEGF-A secretion in conditioned medium (CM) after regorafenib and SC-78 treatment. As shown in Fig. 5A, SC-78 showed a more potent anti-VEGF secretion effect than regorafenib in both MDA-MB-231 and MDA-MB-468 cells. In previous studies, VEGF-A secretion has been shown to bind with VEGF receptor Neuropilin-1 (NRP-1) resulting in forcing breast cancer cell migratory ability^10^,^32^,^33^. Based on immunoprecipitation assay, regorafenib and SC-78 decreased the binding of secreted-VEGF-A to NRP-1 (Fig. 5B, *top*). In addition, the binding of VEGF-A and NRP-1 increased after VEGF_165_ recombinant protein was re-added to the CM (Fig. 5B, *bottom*). We also performed immunofluorescence assay to further observe the binding between secreted-VEGF-A and NRP-1 (Fig. 5C). Also, treatment with VEGF_165_ bioactive recombinant protein enhanced MDA-MB-231 cell migration and rescued the anti-migratory ability induced by regorafenib and SC-78 in both TNBC cell lines (Fig. 5D). These data indicated that regorafenib and SC-78 reduced VEGF-A production and secretion, resulting in decreasing the binding of VEGF-A/NRP-1 and further affecting TNBC cell migration. Since the paracrine interaction of VEGF between vascular endothelial cells and cancer cells are also important for tumorigenesis^34^, we postulated that treatment with regorafenib or SC-78 should also disrupt the paracrine effect of VEGF-A. We collected the CM harvested from MDA-MB-231 and MDA-MB-468 cells exposed to regorafenib or SC-78, and further incubated the human endothelial cells (EAhy926) with the aforementioned CM and analyzed the tube formation of EAhy926 cells after incubation for 24 h. The CM harvested from SC-78 exposed cells had a stronger suppressive effect than that from regorafenib exposed cells on the tube formation ability of human endothelial cells (Fig. 5E). Moreover, we performed a direct *in vivo* angiogenesis assay (DIVAA) to assess the *in vivo* inhibitory effects of SC-78 or regorafenib on human endothelial cells (Fig. 5F). Similarly, the CM harvested from SC-78 exposed MDA-MB-231 cells could decrease the vascular formation *in vivo* more significantly than that from regorafenib exposed cells (Fig. 5F). These results indicated that SC-78 and regorafenib suppressed the secretion of VEGF-A of TNBC cells, and inhibited the paracrine interaction of VEGF-A between TNBC and vascular endothelial cells.

### Anti-cancer effect of regorafenib and SC-78 *in vivo*

**Figure 6:**
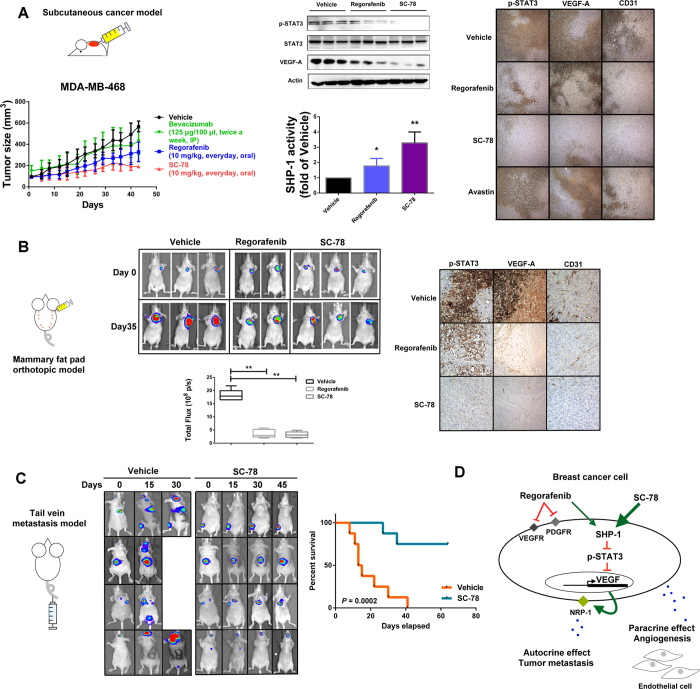
Anti-tumor activity of regorafenib and SC-78 in a murine TNBC metastasis model. (**A**) Nude mice were subcutaneously injected with MDA-MB-468 cells (2 × 10^6^). Mice were treated with vehicle, bevacizumab (125 μg/100 μl, twice a week, IP), regorafenib, or SC-78 (10 mg/kg, everyday, oral) for 43 days (n = 6), and the tumor size (*left, bottom*) was measured. *In vivo* protein levels were analyzed (*Middle, top*). *Middle, bottom,* the *in vivo* SHP-1 activity. *Right,* Representative images of IHC staining (100 × ). *p < 0.05, **p < 0.01. (**B**) Luciferase-expressing MDA-MB-231 (1 × 10^6^) cells were injected orthotopically into the mammary fat pad of the mice. After two weeks, mice received regorafenib and SC-78, or vehicle orally at 10 mg/kg/every day (n = 5). Tumor growth was monitored by IVIS imaging system at the indicated times. *Left*, *top*, visualized by IVIS analysis. *Left, bottom,* quantification analysis from the IVIS total flux. *Right,* Representative images of IHC staining (100×). *p < 0.05, **p < 0.01. (**C**) *In vivo* bioluminescence images of nude mice injected i.v. with MDA-MB-231/*Luc2* cells (1 × 10^6^). After bioluminescence was observed, mice received vehicle or SC-78 orally at 10 mg/kg/every day. *left,* visualized by IVIS analysis. *Right,* Kaplan–Meier plot showing animal survival after treatment with vehicle or SC-78 (n = 8). The survival endpoint was set at 64 days after drug administration. (**D**) Schematic displays the drug mechanism of regorafenib and SC-78 on VEGF-A autocrine and paracrine inhibition and cell migration. SC-78 suppressed cancer metastasis dominantly through SHP-1 dependent-STAT3 dephosphorylation.

To investigate the *in vivo* anti-cancer effects of regorafenib and SC-78 in TNBC, we used three animal models. First, we used subcutaneous xenograft model by injecting MDA-MB-468 cells into nude mice. SC-78 displayed a better antitumor effect on TNBC xenografts compared to regorafenib and bevacizumab (Fig. 6A, *left*). In agreement with our *in vitro* results, SC-78 inhibited the protein expressions of p-STAT3 more potently than regorafenib (Fig. 6A, *middle, upper panel*). In addition, IHC staining with CD31 showed that SC-78 had a more potent effect on decreasing the formation of blood vessels than regorafenib treatment in MDA-MB-468 tumors (Fig. 6A, *right*). Furthermore, the enhancement of SHP-1 activity in TNBC tumors could also be observed more significantly in SC-78-treated groups (Fig. 6A, *middle, lower panel*). These results implied that the regulation of the SHP-1/p-STAT3/VEGF-A pathway is critical for the antitumor effect of SC-78 on TNBC cells. Next, we established an orthotopic model using luc2-expressing MDA-MB-231 cells. The cells were implanted into the mammary fat pad and the tumor burden was analyzed by bioluminescence technology. Representative IVIS images indicated that the SC-78 and regorafenib-treated groups displayed significantly lowered bioluminescence intensity compared to the control group (Fig. 6B, *left*). IHC staining of these orthotopic tumors confirmed that SC-78 and regorafenib downregulated the expressions of p-STAT3, VEGF-A, and the endothelial cell marker CD31 (Fig. 6B, *right*). The results supported that SC-78 and regorafenib suppressed tumor growth and angiogenesis. Thirdly, we generated a tail vein injection metastasis model to evaluate the anti-metastatic effect of SC-78 by injecting luc2-expressing MDA-MB-231 cells into the tail vein of nude mice (Fig. 6C, *left*). Treatment with SC-78 reduced sites of metastatic signals comparing to vehicle treatment (Fig. 6C, middle). Moreover, mice treated with SC-78 showed significantly better survival post tumor cells injection as compared with mice treated with vehicle (Fig. 6C, *right*). Figure 6D summarizes that regorafenib, and its analogue SC-78 devoid of VEGFR/PDGFR kinase inhibition, suppression of metastatic potential of TNBC cells by disruption of the p-STAT3 mediated autocrine and paracrine feed-forward loops of VEGF-A signaling through targeting SHP-1/p-STAT3/VEGF-A axis.

Since clinically the combination of bevacizumab with paclitaxel is still an option for patients with metastatic breast cancer, we further tested SC-78 in combination with paclitaxel, in comparison with bevacizumab plus paclitaxel in MDA-MB-453 and MDA-MB-436 cells. As shown in Supplement Figure S2, the combination of SC-78 with paclitaxel showed comparative anti-migratory effects, in association with p-STAT3/VEGF-A downregulation.

## Discussion

In clinical observation, TNBC behaves more aggressively and increased risk of distant recurrence compared with non-TNBC patients^35^,^36^,^37^,^38^,^39^. We profiled VEGF-A levels in a TNBC patient cohort, and further found similar associations with DMFS and DFS, revealing that risk of developing metastatic disease in TNBC is related to high VEGF-A levels. However, the mechanism resulting in higher VEGF expression in TNBC cells remains unclear. Here, we demonstrated for the first time that VEGF-A expression is reversely correlated with SHP-1 and positively correlated with STAT3 phosphorylation in human TNBC cell lines and clinical patients. We further showed that regorafenib decreased the expression of VEGF-A through the SHP-1/p-STAT3 pathway, hence inhibiting the migration and metastasis abilities of TNBC cells. Importantly, we demonstrated that SC-78, an analog of regorafenib, exhibits better efficacy in decreasing the expression of p-STAT3 and VEGF-A, and also inhibited the metastatic abilities of TNBC cells at relatively low dosage compared to regorafenib. To our surprise, although SC-78, a chemical modification of regorafenib synthesized by deleting the acetyl-pyridine ring, lost the interaction with ATP binding pocket of kinases, it was able to increase SHP-1 activity more than regorafenib. The simple synthetic strategy of SC-78 provides a feasible step for drug development. Given the fact that bevacizumab and regorafenib are both approved for patients with metastatic CRC, we tested SC-78 in combination with 5-FU, in comparison with bevacizumab plus 5-FU, on effects of anti-migration in a panel of CRC cell lines to further support our findings of SC-78 as an effective SHP-1 agonist targeting STAT3/VEGF-A inhibition (Supplement Figure S3). We observed only a non-significant trend in the correlation between SHP-1 and DMFS or DFS in our TNBC patients (Supplement Figure S4). It raises several possibilities: first, our IHC analysis is limited by sample size and most of patients (88/98) displayed lower SHP-1 expression pattern. Secondly, the regulatory network of SHP-1 with other molecules (tumor suppressors or oncoproteins) that might influence patient prognosis remains to be defined. Moreover, biologically the activity of SHP-1 strongly depends on its structure conformation change (open and close forms), such as ligand and chemicals binding and thus expression levels not necessarily correlated with SHP-1 activities^24^,^25^,^26^,^27^; however, it is difficult to directly measure SHP-1 activity from archived patient tumor samples. Nevertheless, more studies are necessary to determine the significance of SHP-1 expression in correlation with survival outcome in patients with TNBC.

In a previous report, VEGF and SEM3A regulate the migration of breast carcinoma against the autocrine NRP-1 ligands^32^. Here, we demonstrated that both regorafenib and SC-78 could decrease the endogenous level of VEGF-A and the inhibition of secreted VEGF-A would further reduce the binding of VEGF-A to receptor NRP-1, which might lead to increased binding of SEMA3A and NRP-1 and reduce the migration of breast cancer cells. VEGF is also known to be involved in fibroblast proliferation and immune cell infiltration in the tumor microenvironment, both of which are essential for development of tumor malignancy^40^. Therefore, whether regorafenib and SC-78 can affect the interaction between tumor cells and other stromal populations warrants our further investigation.

Our *in vivo* model results show that bevacizumab alone is less effective in suppressing TNBC tumor growth comparing with regorafenib or SC-78. Clinically, bevacizumab have shown only modest success in breast cancer patients^12^,^13^,^14^. However, recent subgroup analyses have suggested beneficial use of bevacizumab in combination with chemotherapy in TNBC subpopulation in terms of trend toward better PFS and OS^41^,^42^,^43^. Preclinical studies have shown tumor regrowth with ongoing anti-angiogenic treatment or rebound angiogenic activity after withdrawal of antiangiogenic therapy^44^,^45^,^46^. Our results suggested that targeting SHP-1/p-STAT3 dependent VEGF-A suppression might be an effective approach to suppress metastasis. To date, among several VEGFR multi-targeted TKIs such as sunitinib, sorafenib, and vandetanib that were tested in clinical trials, only sorafenib has resulted in encouraging combination data in metastatic breast cancer, with modest improvements in PFS^34^,^47^. It is possible that the type of anti-angiogenic agent is critical and more studies are necessary to confirm the optimal approach for suppressing metastasis via VEGF-dependent strategies.

In summary, our study has provided new insights on the mechanisms of anti-angiogenesis and anti-metastasis by demonstrating for the first time that SHP-1/STAT3 is one of the regulatory pathways of VEGF-A in human TNBC cells. Importantly, this study indicates that SC-78 is a promising small molecule for VEGF-A inhibition, and is a potential therapeutic lead candidate for TNBC patients.

## Methods

### Cell Culture

All cell lines were obtained from American Type Culture Collection (ATCC, Manassas, VA), and were immediately expanded and frozen down such that all cell lines could be restarted every 3 months from a frozen vial of the same batch of cells.

### Conditioned Medium (CM)

Cells were first seeded onto 100 mm dishes at a density of 1 × 10^6^ cells/dish for 24 h. After incubation of drugs for 24 h, the medium was replaced with 5 ml of fresh medium with 1% FBS and incubated for another 24 h. Then the CM was harvested, centrifuged at 3,000 rpm for 10 min to remove the cell debris, and stored at -80°C until use.

### Antibodies

p-STAT3 and STAT3 were from Cell Signaling (Danvers, MA). SHP-1, NRP-1 antibodies were purchased from Abcam (Cambridge, MA). VEGF-A antibody (NB100-664) was purchased from Novus Biologicals.

### Gene knockdown using siRNA

Smart-pool small interfering RNAs (siRNAs), including the control (D-001810-10), SHP-1, were purchased from Dharmacon (Chicago, IL). The knockdown procedure was as described previously^48^.

### Wound healing assay

A confluent monolayer was disrupted using a 0.5-mm cell scraper in culture dishes, washed with PBS, and then incubated for 24 h in medium with 5% FBS. The wound area were measured by aid of Image Pro-Plus software (Media Cybernetics Inc., Bethesda, MD, USA)

### Transwell Migration Assay

The Boyden chamber system was used to evaluate cell migration. After drug treatment for 24 h, 5 × 10^3^ cells suspended in 100 μl of 1% FBS medium were seeded in the upper chamber of a Transwell plate (Corning-Costar, Corning, NY; 8-μm pore size). The lower chamber was filled with medium containing 10% FBS. After incubation for 16 h, the cells on the top side of the upper Transwell membrane were removed using cotton swabs. The cells trapped on the bottom side of the membrane were fixed with methanol and stained with a 4′,6-diamidino-2-phenylindole solution (10 μg/ml; Invitrogen). The number of cells from eight different fields on each membrane was counted under a fluorescence microscope.

### Tube Formation Assay

A 96-well plate was precoated with Matrigel for 1 h before seeding EA.hy926 hybrid endothelial cells (5 × 10^4^ cells/well) at 37°C. Tube formation ability was then assayed by quantifying the complexity of the tubes formed using KURABO Angiogenesis Image Analyzer software^49^.

### Evaluation of VEGF-A expression

Expression of VEGF-A was assessed semiquantitatively via an immunohistochemical score (H score), as defined by multiplying the percentage of positive carcinoma cells (0–100) by the staining intensity (0–3), and this gave an H score ranging from 0 to 300^50^. Staining intensity was given four grades: none (0), weak (1), moderate (2) and strong (3). The chosen cut-off value is similar to those used in previous studies^51^.

### Directed *in Vivo* Angiogenesis Assay (DIVAA)

*In vivo* angiogenesis assays were performed using a DIVAA kit (Trevigen). In brief, silicon angioreactors were filled with basement membrane extracts containing CM and heparin (1 μmol/liter), and these angioreactors were subcutaneously implanted into both flanks of female NCr athymic nude mice. After 17 days, the reactors were excised and labeled with FITC-lectin. The fluorescence intensity was measured by microplate reader.

### SHP-1 phosphatase activity

A RediPlate 96 EnzChek Tyrosine Phosphatase Assay Kit (R-22067) was used for SHP-1 activity assay (Molecular Probes, Carlsbad, CA). The method was as described previously^52^.

### Quantitative real time PCR (QPCR)

qPCR was performed as described by Ponchel *et al.*^53^. Oligonucleotide sequences were as follows: VEGF-A, 5′-CTTGCCTTGCTGCTCTACC-3′ (forward) and 5′-CACACAGGATGGCTTGAAG-3′ (reversed); GAPDH, 5′-CGACCACTTTGTCAAGCTCA-3′ (forward) and 5′-AGGGGTCTACATGGC AACTG-3′ (reversed). Thermocycling was performed by Roche LightCycler 480 sequence detection system (Roche Applied Science, Foster, California). Expression levels of genes of interest were normalized to that of GAPDH in the same sample.

### Chromatin immunoprecipitation (CHIP) assay

CHIP assay was performed according to the protocol provided with the EZ ChIP chromatin immunoprecipitation and EZ-Zyme Chromatin prep kit (Upstate Biotechnology, Lake Placid, NY). The genomic DNA was extracted and fragmented into approximately 200-bp fragments using the EZ-Zyme kit after crosslinking (Upstate Biotechnology, Inc., Lake Placid, NY). Each reaction mixture was incubated with 2 μg STAT3 antibodies or nonspecific rabbit IgG (Millipore). The immunoprecipitated products were washed and eluted sequentially according to the manufacturer’s instructions. Finally, the precipitated DNA was recovered using the spin column that was provided in the kit. The sequences of the primers used in the ChIP assay were as follows: 5′-GGTTTTGCCAGACTCCACAGTGC-3′, and 5′- GTGTGTCCCT CTGACAATGTGCC-3′.

### Construction of VEGF-A promoter

The VEGF-A promoter (−2024 bp to +46 bp) was amplified using human genomic DNA as the template. The amplified product was digested with *Hind*III and *Xho*I and subsequently cloned into pGL4-basic vector. The deletion fragments were made by PCR amplification using different 5′ deletion primers with a 5′*Xho*I restriction site and the same 3′ primer and were ligated to the pGL4 basic vector in the multiple cloning sites between *Hind*III and *Xho*I and DNA sequences were confirmed.

### Mutation of STAT3 binding site on VEGF-A promoter

The mutagenic constructs were made using the QuickChange II XL Site-Directed Mutagenesis Kit (Stratagene). For this, the mutagenic primers with mutations in STAT3 binding site were synthesized by the MDBio. The sequences were as follow: F: 5′-CCCTGGACACTTCCGTTAGGACCCCAGT-3′, and R: 5′-ACTGGGGTCCTAACGGAAGTGTCCAGGG-3′.

### Dual luciferase assay

After transfection with firefly luciferase reporter construct and reference pCMV-renilla luciferase plasmid for 48 h, the promoter activity was analyzed by dual luciferase assay according to the manufacturer’s protocol.

### Immunofluorescence

Cells grown on coverslips were washed with cold PBS, fixed with ice-cold methanol, and permeabilized with 0.5% (v/v) Triton X-100. Coverslips were incubated with primary antibodies against human VEGF-A and NRP-1 for 1 h at 37°C, washed with PBST, and subsequently incubated with rabbit secondary antibody conjugated with rhodamine for NRP-1 and mouse secondary antibody conjugated with FITC for VEGF-A. Afterwards, the coverslips were washed and fluorescence images were taken with a Zeiss LSM 510 confocal microscope.

### Immunohistochemistry (IHC)

Tumors were sectioned and then stained with indicated antibodies. The standard IHC staining was then performed according to the manufacturer’s instructions of LSAB2 streptavidin-biotin complex system (Dako Corp, Carpinteria, CA).

### *In silico* survival analysis using a publically available database

The Kaplan Meier plotter is online survival analysis software (Accessible at http://kmplot.com/analysis/) to assess the prognostic value of biomarkers using transcriptomic data^54^,^55^,^56^. Accordingly, a background database was established using gene expression data and survival information of patients downloaded from GEO (Affymetrix HGU133A and HGU133 + 2 microarrays). As of the year 2014, a total of 4,142 breast, 1,648 ovarian, 2,437 lung and 765 gastric cancer patients with a mean follow-up of 69/40/49/33 months were included in this database. The primary purpose of this tool is a meta-analysis-based biomarker assessment^54^,^55^,^56^. Using the selected parameters from the website, survival data and curves according to specific genes (mRNA expression) can be auto-generated. For survival analysis of VEGF-A gene expression, auto select best cutoff was chosen and Affymetrix probe ID 210512 was chosen based on default suggestion by website.

### Animal studies

Female NCr athymic nude mice (5–7 weeks of age) were obtained from the National Laboratory Animal Center (Taipei, Taiwan). For the subcutaneous mouse model, MDA-MB-468 tumors reached 100 mm^3^, mice received regorafenib, SC-78, or bevacizumab. Controls received vehicle. For orthotopic animal studies, mice were inoculated into the 2^nd^ mammary fat pad directly with MDA-MB-231/luc2 cells. Once the tumors were visible, mice were separated into 3 groups (vehicle, regorafenib, and SC-78). Tumor infiltration was monitored by bioluminescence imaging. For tail vein metastasis model, MDA-MB-231/luc2 cells were injected into the tail vein of 5-week-old female nude mice. Mice were randomly grouped into SC-78-treated or untreated groups when cells were initially observed the luminescence signals. Drug treatment persisted until mice died or 64 days. All experiments were performed in accordance with the Institutional Animal Care and Use Committee of Taipei Veterans General Hospital. All experimental protocol involving mice were approved by the ethical committee of Taipei Veterans General Hospital and performed in accordance with approved guidelines and regulations.

### Breast tumor tissue from patients with TNBC

Paraffin-embedded tissue microarray of tumor samples from TNBC patients sections (4-μm) on poly-1-lysine-coated slides were prepared for IHC experiments. Patients’ general characteristics were collected at time of tumor sample collection, including age, AJCC (TNM) tumor stage. Survival status and events according to recurrence (local and distant metastasis) were followed till date of last follow-up. This study was approved by the ethics committee of Institutional Review Board of Taipei Veterans General Hospital. All informed consents from sample donors were in accordance with the Declaration of Helsinki and were obtained at time of their donation. All methods were conducted in accordance with the relevant guidelines.

### Statistical analysis

Statistical comparisons were based on nonparametric tests and statistical significance was defined as a P value of less than 0.05. All statistical analyses were performed using SPSS for Windows version 12.0 software (SPSS, Chicago, IL). In clinical data, categorical variables were compared by chi-square test, and the prognostic impact of VEGF-A IHC score (H-score) on DFS and DMFS were analyzed by cox regression and adjusted with tumor staging (AJCC staging). Data are expressed as mean ± SD or SE.

## Additional Information

**How to cite this article**: Su, J.-C. *et al.* Disrupting VEGF-A paracrine and autocrine loops by targeting SHP-1 suppresses triple negative breast cancer metastasis. *Sci. Rep.* **6**, 28888; doi: 10.1038/srep28888 (2016).

## Supplementary Material

Supplementary Information
