# Supplementary material for: Disrupting VEGF-A paracrine and autocrine loops by targeting SHP-1 suppresses triple negative breast cancer metastasis
Source: Sci Rep. 2016 Jul 1;6:28888. doi: 10.1038/srep28888 (PMC4929457; doi:10.1038/srep28888)
Supplement: Supplementary Information [file srep28888-s1.pdf]

Disrupting VEGF-A paracrine and autocrine loops by targeting SHP-1 suppresses triple negative breast cancer metastasis

Jung-Chen Su[1,2,] , Ai-Chung Mar[10], Szu-Hsien Wu[1], Wei-Tien Tai[3, 4], Pei-Yi Chu[5,6], Chia-Yun Wu[7,8], Ling-Ming Tseng[8,9], Te-Chang Lee[11], Kuen-Feng Chen[3,4], Chun-Yu Liu[7,8,*] , Hao-Chieh Chiu[2,*] and Chung-Wai Shiau[1,*]

Supplementary Figure Legends

Figure S1. Kaplan-Meir survival analysis of VEGF-A mRNA in 249 TNBC patients as obtained using the Kaplan-Meier Plotter (http://kmplot.com/analysis/index). The disease-free survival was shorter in the VEGF-A mRNA high expression group than in the VEGF-A mRNA low expression group, *P* < 0.001. Auto select best cutoff was chosen in the analysis; cutoff value used was 6025; expression range of the probe (Affymetrix probe ID 210512) was 223–19641.

Figure S2.The anti-migratory effects of paclitaxel combined with bevacizumab (*left*) or SC-78 (*middle*). MDA-MB-453 (A) and MDA-MB-436 (B) cells exposed to the indicated treatments were analyzed by Transwell migration assay. *Right*, Two cell lines exposed to SC-78 in various dosages or combined with paclitaxel 60 nM were analyzed by western blot. Representative western blot images of three identical experiments are shown.

Figure S3. The anti-migratory effects of 5-FU combined with bevacizumab (*left*) or SC-78 (*middle*). Four different colorectal cancer cells (RKO (A), RKO-E6 (B), H3347(C), HCT116 (D)) exposed to the indicated treatments were analyzed by Transwell assay. *Right*, Cells exposed to SC-78 in various dosages or combined with 5-FU 5 μM were analyzed by western blot. Representative western blot images of three identical experiments are shown.

Figure S4. Kaplan-Meir survival analysis of SHP-1 expression in 98 TNBC patients. Kaplan-Meier graph was prepared to compare DFS (*left*) and DMFS (*right*) in patients with high SHP-1 (H score >= 300) or low SHP-1 (H score < 300) levels for the indicated time of follow up. Chi-square test indicated a significant difference between SHP-1 high (N = 10) and low (N = 88) patients.

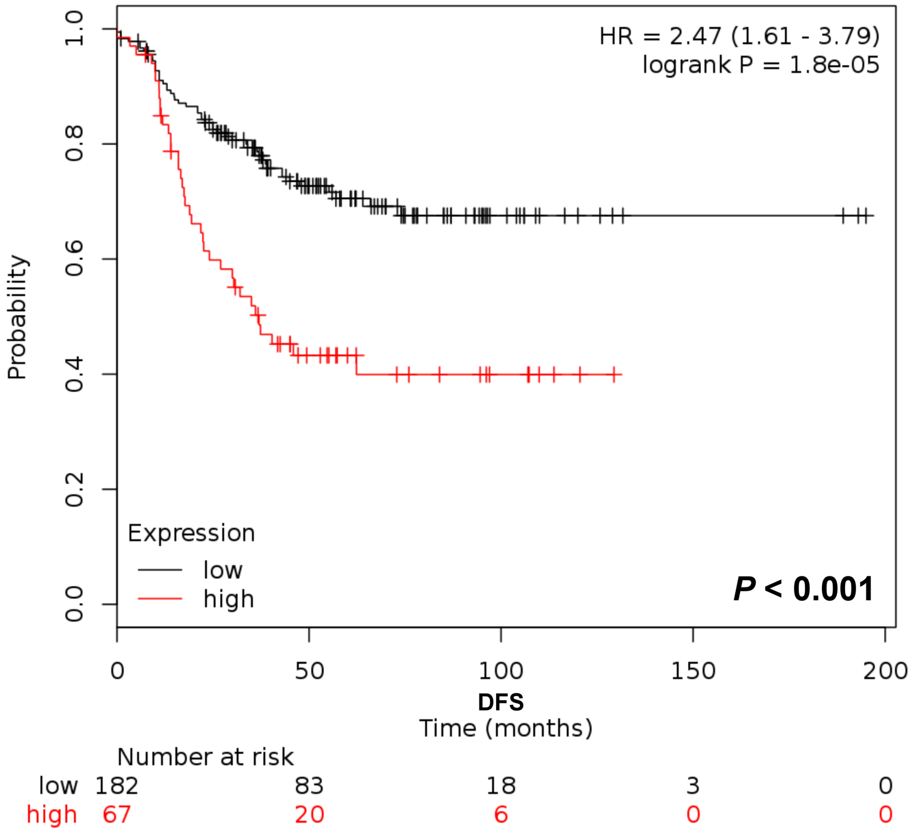
HR = 2.47 (1.61 - 3.79)
logrank P = 1.8e-05
Probability
Expression
low
high
P < 0.001
DFS
Time (months)
Number at risk
low 182 83 18 3 0
high 67 20 6 0 0

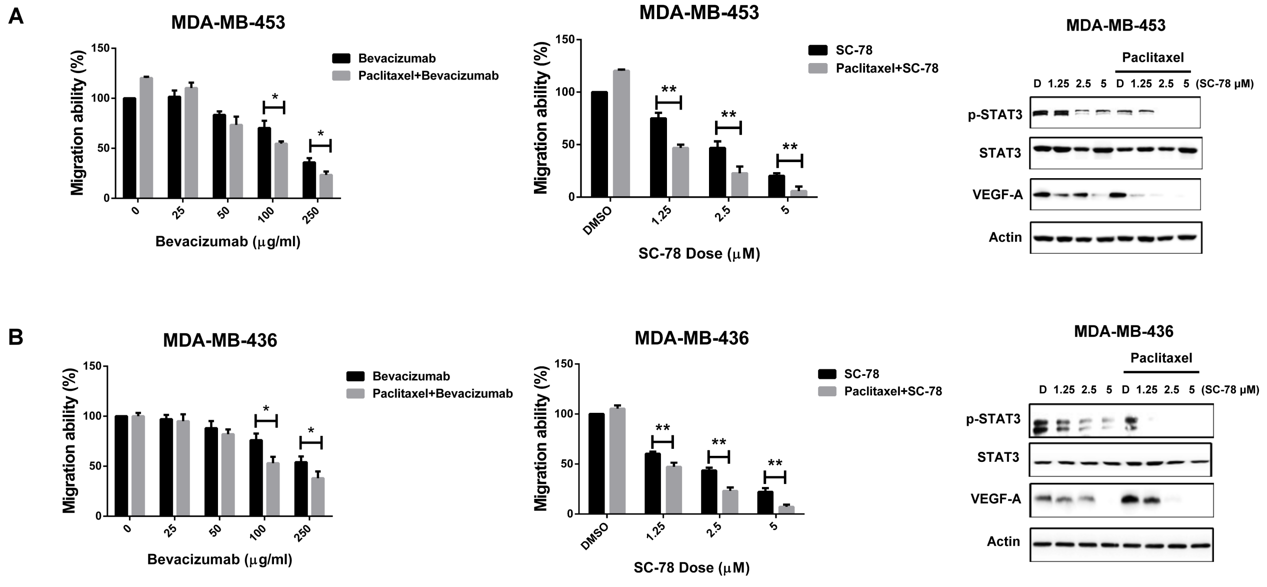
A
MDA-MB-453
Migration ability (%)
Bevacizumab
Paclitaxel+Bevacizumab
Bevacizumab (μg/ml)
MDA-MB-453
SC-78
Paclitaxel+SC-78
SC-78 Dose (μM)
DMSO
MDA-MB-453
Paclitaxel
D 1.25 2.5 5 D 1.25 2.5 5 (SC-78 μM)
p-STAT3
STAT3
VEGF-A
Actin
B
MDA-MB-436
Migration ability (%)
Bevacizumab
Paclitaxel+Bevacizumab
Bevacizumab (μg/ml)
MDA-MB-436
SC-78
Paclitaxel+SC-78
SC-78 Dose (μM)
DMSO
MDA-MB-436
Paclitaxel
D 1.25 2.5 5 D 1.25 2.5 5 (SC-78 μM)
p-STAT3
STAT3
VEGF-A
Actin

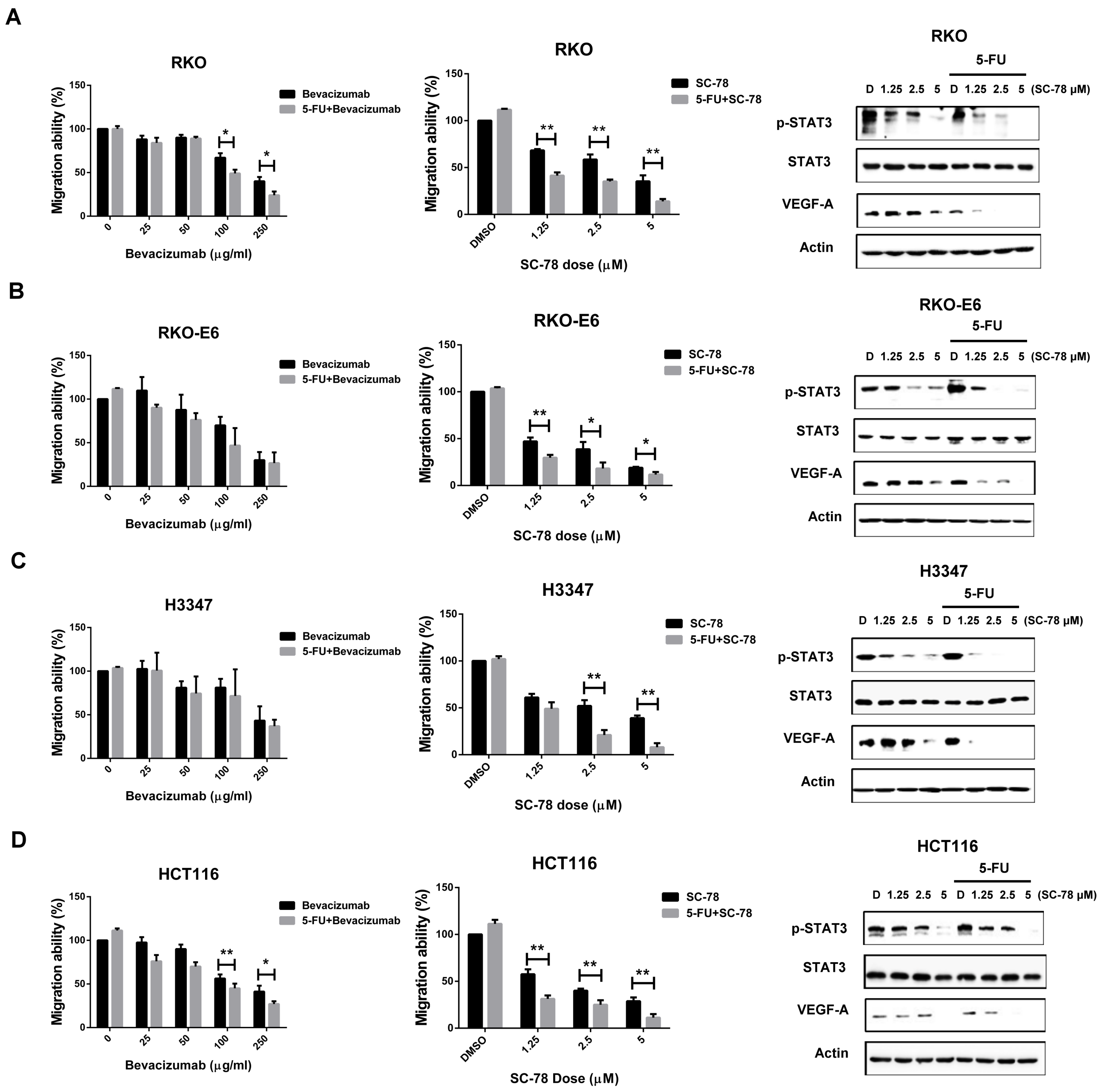

A
RKO
Migration ability (%)
Bevacizumab
5-FU+Bevacizumab
Bevacizumab (μg/ml)
RKO
SC-78
5-FU+SC-78
SC-78 dose (μM)
DMSO
RKO
5-FU
D 1.25 2.5 5 D 1.25 2.5 5 (SC-78 μM)
p-STAT3
STAT3
VEGF-A
Actin
B
RKO-E6
Migration ability (%)
Bevacizumab
5-FU+Bevacizumab
Bevacizumab (μg/ml)
RKO-E6
SC-78
5-FU+SC-78
SC-78 dose (μM)
RKO-E6
5-FU
D 1.25 2.5 5 D 1.25 2.5 5 (SC-78 μM)
p-STAT3
STAT3
VEGF-A
Actin
C
H3347
Migration ability (%)
Bevacizumab
5-FU+Bevacizumab
Bevacizumab (μg/ml)
H3347
SC-78
5-FU+SC-78
SC-78 dose (μM)
H3347
5-FU
D 1.25 2.5 5 D 1.25 2.5 5 (SC-78 μM)
p-STAT3
STAT3
VEGF-A
Actin
D
HCT116
Migration ability (%)
Bevacizumab
5-FU+Bevacizumab
Bevacizumab (μg/ml)
HCT116
SC-78
5-FU+SC-78
SC-78 Dose (μM)
HCT116
5-FU
D 1.25 2.5 5 D 1.25 2.5 5 (SC-78 μM)
p-STAT3
STAT3
VEGF-A
Actin

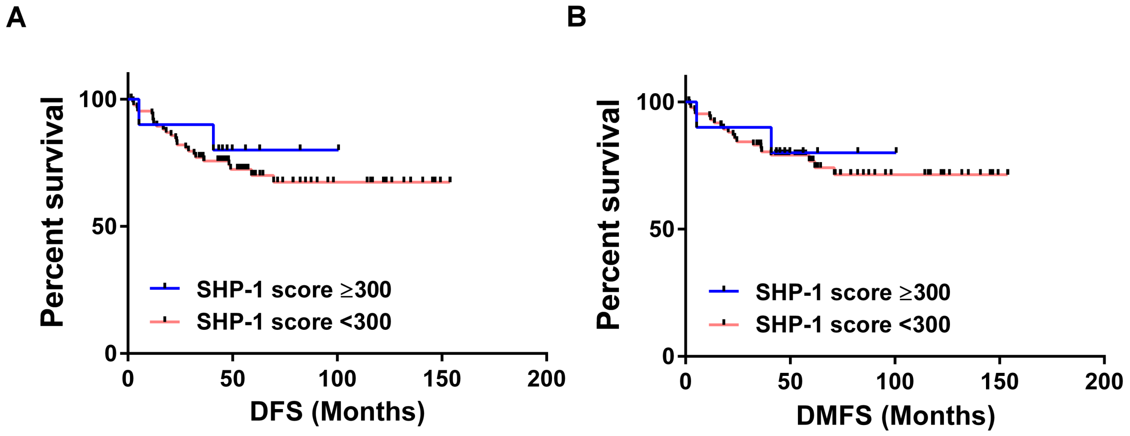
A
Percent survival
100
50
0
SHP-1 score ≥300
SHP-1 score <300
0
50
100
150
200
DFS (Months)
B
Percent survival
100
50
0
SHP-1 score ≥300
SHP-1 score <300
0
50
100
150
200
DMFS (Months)

Disrupting VEGF-A paracrine and autocrine loops by targeting SHP-1 suppresses triple negative breast cancer metastasis

Jung-Chen Su[1,2,], Ai-Chung Mar[10], Szu-Hsien Wu[1], Wei-Tien Tai[3, 4], Pei-Yi Chu[5,6], Chia-Yun Wu[7,8], Ling-Ming Tseng[8,9], Te-Chang Lee[11], Kuen-Feng Chen[3,4], Chun-Yu Liu[7,8,*], Hao-Chieh Chiu[2,*] and Chung-Wai Shiau[1,*]

Supplementary Table S1. Clinical significance of VEGF-A expression in patients with primary triple-negative breast cancers

	**High VEGF-A H-score (>160) N = 21**	**Low VEGF-A H-score (<=160) N = 76**	**P value**
**Age, median (range)**	55 (36-75)	55.5 (35-88)	0.972
**AJCC Stage***			0.957
**0**	0 (0.0%)	1 (1.3%)	
**1**	4 (19.0%)	13 (17.1%)	
**2**	12 (57.1%)	44 (57.9%)	
**3**	5 (23.8%)	18 (23.7%)	
**Disease recurrence**	11 (52.4%)	15 (19.7%)	0.003
**Distant metastasis**	10 (47.6%)	12 (15.8%)	0.002
**Local recurrence**	5 (23.8%)	9 (11.8%)	0.167

Cox regression analysis of DFS

	**Univariate**			**Multivariate**		
	HR	95% CI	P value	HR	95% CI	P value
**AJCC Stage**	3.33	1.72-6.44	<.001	3.45	1.79-6.68	<.001
**VEGF-A H score >160**	3.21	1.47-7.03	.003	3.45	1.57-7.55	.002

HR, hazard ratio; CI, confidence interval; DFS, disease-free survival.
H-score, histology score as determined by immunohistochemical staining described in Material and Methods.
*American Joint Committee on Cancer (AJCC) staging is based on the 7th edition[1].

References of Supplementary Table S1:

1 Edge, S. B. & Compton, C. C. The American Joint Committee on Cancer: the 7th edition of the AJCC cancer staging manual and the future of TNM. *Annals of surgical oncology* **17**, 1471-1474, doi:10.1245/s10434-010-0985-4 (2010).
